# Undetected *Plasmodium malariae* and *P. ovale* infections in HRP2 RDT-positive children with uncomplicated malaria in Nanoro, Burkina Faso

**DOI:** 10.5281/zenodo.15965746

**Published:** 2025-07-16

**Authors:** Amélé Fifi Chantal Kouevi, Ipéné Mylène Carenne Bayala, Paul Sondo, Bérenger Kaboré, Kié Solange Millogo, Sié A. Elisée Kambou, Eulalie W. Compaore, Moustapha Nikiema, Adama Kazienga, Toussaint Rouamba, Awa Gnémé, Halidou Tinto

**Affiliations:** 1 Institut de Recherche en Sciences de la Santé (IRSS)/ Clinical Research Unit of Nanoro (CRUN), Nanoro, Burkina Faso.; 2 Laboratoire de Biologie et Ecologie Animales/ Université Joseph KI-ZERBO, Ouagadougou, Burkina Faso.

## Abstract

**Background:**

The widespread use of histidine-rich protein 2 (HPR2)-based rapid diagnostic tests (RDTs), specific to *Plasmodium falciparum* in endemic areas may underestimate the weight of minor species such as *P. malariae* and *P. ovale* in malaria transmission. This study aimed to determine the extent of undetected *P. malariae* and *P. ovale* infections in children with positive diagnosis of uncomplicated malaria based on HRP2 RDT in the Nanoro health district, Burkina Faso.

**Materials and Methods:**

Children <5 yrs with uncomplicated malaria confirmed by HRP2 RDT were recruited from July 2021 to June 2022 in five peripheral health facilities of the Nanoro health district. Blood samples were collected from finger prick for malaria species identification by microscopy and nested PCR. The prevalence of *P. malariae*, *P. ovale*, and mixed infections was estimated as the ratio of positive cases over the total samples analysed. Binomial generalised linear models were used to assess the effect of age and sex on the positivity rate of mixed infections.

**Results:**

Over the study period, 207 children with uncomplicated malaria who tested positive for the HRP2 antigen were included. Microscopy detected 4 non-*falciparum* cases: 3 *P. malariae* and 1 *P. ovale*. In addition to these patent cases, sub-patent infection with *P. ovale* and *P. malariae* were detected in 6 and 5 cases, respectively. Mixed infections with non-*falciparum* species exhibited lower parasite densities than mono-infections with *P. falciparum* alone. There was no effect of gender or age on the mixed infection positivity rate (X^2^=0.16, p=0.683).

**Conclusion:**

The widespread use of HPR2-based RDTs underestimate the burden of non-*falciparum* species. In the context of eliminating malaria, new diagnostic tools allowing the detection of *Plasmodium* species other than *P. falciparum* must be deployed.

## Introduction

The malaria burden in children <5 yrs remains high in sub-Saharan Africa, despite multiple interventions programmes [1]. In 2023, the African region accounted for over 94% of global cases, with 246 million infections and 569,000 deaths [2]. In Burkina Faso, malaria remains the leading cause of medical consultations and hospitalisations. In 2023 the country recorded 9,731,712 malaria cases, resulting in 3,385 deaths, including 2,216 children <5 yrs [3].

Over the past decades, various interventions that specifically target children <5 yrs have been implemented, like seasonal malaria chemo-prevention (SMC) as part of global efforts to eliminate malaria. The World Health Organization’s (WHO) unified guidelines consolidate all recommendations relating to malaria prevention, diagnosis, treatment, and elimination [4]. Among these strategies, systematic diagnosis and management of all suspected malaria cases are implemented. For this purpose, WHO recommends the use of microscopic examination (thick and thin blood smears) and rapid diagnostic tests (RDTs) as diagnostic tools [5]. The latter relies on the detection of specific antigens such as histidine-rich protein 2 (HRP2), lactate dehydrogenase (LDH), or aldolase [6,[7]. HRP2 is a specific marker for *Plasmodium falciparum*, whereas aldolase is common to all *Plasmodium* species [5,[6,[8].

The introduction of RDTs has significantly improved access to malaria diagnosis, particularly in rural settings where access to quality microscopy remains limited [5,[9]. Their ease of use, affordability, and availability in the field make them an essential tool in national malaria control strategies [4,[10]. Nevertheless, the most widely used RDTs specifically designed to detect *P. falciparum*, the species responsible for the vast majority of malaria cases, raises concerns about the underestimation of malaria infections caused by other species, particularly *P. malariae* and *P. ovale* [11,[12]. This issue is even more concerning in endemic areas where mixed infections involving multiple *Plasmodium* species are common [13–15].

The limited sensitivity of the available RDTs for detecting non-*falciparum* species restricts our ability to assess the burden of these non-*falciparum* species and their impact on malaria epidemiology. Although some pan-species RDTs have been developed, there is still insufficient documentation of their performance for the reliable detection of *P. malariae*, *P. ovale*, and *P. knowlesi* [10]. This diagnostic gap highlights the need to improve the diagnostic tools in order to characterise the full spectrum of *Plasmodium* infections, particularly in settings where malaria elimination strategies are underway. Nucleic acid detection techniques, such as the polymerase chain reaction (PCR) and loop-mediated isothermal amplification (LAMP), are highly sensitive and extremely useful for identifying mixed infections. This is particularly important when the parasite density is too low to be detected by standard microscopy or RDTs [4]. The sensitivity and specificity of PCR methods are superior to those of microscopy and RDTs, with the detection limit of nested PCR set at as low as 0.2 parasites/μl [16].

In Burkina Faso, first-line malaria diagnosis in peripheral health facilities relies heavily on RDTs targeting the HRP2 antigen, specific to *Plasmodium falciparum*, the most commonly observed species. While this approach is effective for detecting *P. falciparum*, other *Plasmodium* species, such as *P. malariae* and *P. ovale*, are missed as reported in previous studies [13,[17]. In such a context, the prevalence of these non-*falciparum* infections, particularly among children diagnosed with uncomplicated malaria using only HRP2-based RDTs, remains largely unknown.

Therefore, the present study was initiated to assess the extent of missed *P. malariae* and *P. ovale* infections in children with positive diagnosis of uncomplicated malaria based on HRP2 RDT in the Nanoro health district, Burkina Faso.

## Materials and Methods

The study was carried out in the Nanoro health district (NHD), a rural area of the Central-Western region of Burkina Faso. The NHD is located at approximately 85 km from Ouagadougou. Data collection took place at four health facilities of the department of Soaw (Soaw, Zoetgomdé, Kologkom, and Poessé) and at one health facility (Kokolo) of the department of Nanoro. Malaria transmission is seasonal and hyperendemic, with the majority of cases occurring from July to December, overlapping with the rainy season [18]. In 2023, an estimated 126,294 cases of malaria were reported in the NHD [3]. The most common species is *P. falciparum* which is responsible of about 90% of malaria cases [19].

Samples were collected between July 2021 and June 2022 during the SMC-RST (NCT04816461) study. The aim of the SMC-RST study was to improve the impact of SMC in terms of reducing malaria morbidity and mortality in children <5 yrs through a new strategy consisting of screening and treatment of household members of SMC children. The description of the protocol for this trial has been detailed by Sondo *et al.* [20]. During this trial, thick and thin smears were collected by finger prick for haemoglobin (Hb) measurement, RDT, microscopy, and dried blood spot (DBS) onto filter paper (Whatman n°3, Cytiva, China) for PCR analysis. Each dried blood spot was initially stored in a Ziploc bag with silica gel (desiccant), to preserve the sample's integrity, placed in boxes, and kept at room temperature in the laboratory.

For this report, only children of the control (no intervention) arms of the project were considered. Children who attended the health facilities at least once (unscheduled visit) to seek care with a positive diagnosis of uncomplicated malaria, as determined by HRP2 RDT and microscopy, and having DBS available were included.

Children were classified into two groups regarding *Plasmodium* species as mono-infection or mixed infection according to the number of malaria species detected on the thin film by light microscopy and PCR.

### Sample size estimation

The sample size calculation followed Schwartz’s formula:

N= (Z × P (1-P))/d^2^

where N represents the sample size, P corresponds to the prevalence of mixed infections (5%) [17], Z is the value associated with the chosen confidence level (1.96 for a 5% error risk), and d denotes the desired precision (3%). The estimated sample size was 203. Considering a 5 % non-response rate, the final sample size was adjusted to at least 213 participants.

### Body temperature and haemoglobin measurement

Body temperature was measured using a non-contact clinical thermometer (Microlife NC 200, Switzerland), positioned 1 to 5 cm from the forehead. The temperature was automatically recorded in degrees Celsius.

Screening for anaemia was performed by haemoglobin (Hb) measurement using the analyser HemoCue® 801+ (SOC-HE121916, Danayer group, Angelholm, Sweden). A drop of undiluted blood was placed into a microcuvette, inserted into the device, and the Hb concentration was instantly displayed in g/dL.

### Rapid diagnostic test

The AdvDx™ Malaria Pf test kit (004ADFEF025KI-1, Advy Chemical Pvt. Ltd, India) was used for the rapid diagnosis of malaria. This antigen test targets PfHRP2 of *P. falciparum*. The test was carried out according to the manufacturer’s instructions.

### Microscopic diagnosis

Thick and thin smears were prepared and stained with 3% Giemsa for 30 min and double read by two independent microscopists using an Olympus CX21 microscope (Olympus Corporation, Tokyo, Japan). *Plasmodium* species were identified using thin smears, while parasite density was assessed from thick smears. Parasite densities were estimated by counting asexual parasites against 200 white blood cells on thick smears and calculating parasites per microliter of blood, assuming a standard WBC count of 8,000/μL. A smear was considered negative if no asexual form of the parasite was observed after examining 100 thick-film fields [21]. All microscopists were qualified by the National Institute for Communicable Diseases (NICD, South Africa) [22].

### Molecular analysis

*Plasmodium* DNA was extracted using the tween®20-chelex®100 resin method previously described [23] from randomly selected DBS from positive microscopic samples. Briefly, an approximately 3 mm diameter portion of each DBS was excised and introduced into a 1.5 mL Eppendorf tube, followed by the addition of 1 mL of 0.5% Tween® 20 in 1X phosphate-buffered saline (PBS). This was vortexed and incubated at 4°C for at least 12 hours, after which the supernatant was discarded. Next, 1 ml of 1X PBS was added to each tube, incubated at 4°C for 30 min, and then aspirated and discarded. Subsequently, 150 *μ*L of a 10% Chelex® 100 resin solution was added to each tube, followed by incubation at 95°C for 10 min. During this incubation, each tube was briefly vortexed (5–10 seconds) at least twice. Finally, the tubes were centrifuged at 13,200 rpm for 5 min, and the clear supernatant containing the extracted DNA was collected and transferred to DNA storage tubes for immediate use or storage at -20 °C for later analyses.

The species of *Plasmodium* were identified using a nested PCR approach targeting the 18S small subunit ribosomal DNA, as previously described [24]. Briefly, this method consisted of two PCRs. The first PCR for detecting the *Plasmodium* genus, and the second using the product of the first PCR as DNA template and specific primers to identify the species of *Plasmodium* (*P. falciparum*, *P. vivax*, *P. malariae*, and *P. ovale*). A No Template Control (NTC) was used in all reactions and genomic DNA from laboratory strains or clinical isolates were used as a positive control for respective species.

For the primary standard PCR reaction, 2 *μ*L of genomic DNA was used in a 25 *μ*L reaction with outer primers rPLUf and rPLUr. The PCR assays were performed using a heating block (PTC-100, MJ Research Inc., USA). The cycling parameters for the first amplification reaction were as follows: a primary denaturation for 1 min at 94°C followed by 35 cycles consisting of a 1 min denaturation at 94°C, a 2 min primer annealing step at 58°C, and a 5 min extension step at 72°C. These cycles were followed by a final extension step at 72°C for 5 min and a hold step at 4°C.

The second round was performed with 2 *μ*L of the primary PCR product and species-specific primers for the four human malaria species in separate reaction tubes. The amplification programme remained the same, except for the number of cycles, which was followed by 30 cycles.

The amplification products were then visualised by electrophoresis on a 2.5% agarose gel after staining with ethidium bromide. The electrophoresis was run at 100 volts for 2.5 hrs. Gel imaging was carried out using a trans ultraviolet illuminator (Biotec-Fischer GmbH, Reiskirchen, Germany). The size of the specific PCR product is different for each of the species: 205 bp for *P. falciparum*, 120 bp for *P. vivax*, 144 bp for *P. malariae* and approx. 800 bp for *P. ovale*.

### Statistical analysis

Descriptive statistics were performed using the proportion and geometric mean (95% confidence interval) for qualitative data and parasite density, respectively. Haemoglobin level was used to define anaemia status as no anaemia (Hb ≥ 11 g/dL) and anaemia (Hb < 11 g/dL). Age groups were also defined as <2 and ≥2 yrs. The frequencies of *P. falciparum*, *P. malariae*, *P. ovale*, and mixed infections were estimated as proportions. Binomial generalised linear models were used to assess the effect of age and sex on the positivity rate of mixed infections. The parasite density between mono and mixed-infections was compared using the Mann-Whitney U test. Data were analysed using R version 4.3, and Stata version 14 (StataCorp, College Station, TX, USA), and a p-value of <0.05 was considered statistically significant.

### Ethical approval and informed consent

This study was part of a study that received approval from the Health Research Ethics Committee in Burkina Faso (SMC RST No. 2021-03-059) on 10 March 2021. Written informed consent was obtained from parent(s)/guardian(s) for all participants prior to enrolment in the study. The informed consent interview with parents/guardians was confidential. In case the parents/guardians were illiterate, an impartial and literate witness was present.

## Results

The majority of participants in this study were children >2 yrs of age which accounted for 74.4 % of the study population vs 25.6% for children <2 yrs old (Table 1). About 81.5 % of children presented with fever. Anaemia was high, with 81.55% of children presenting with a haemoglobin level of less than 11 g/dL. The geometric mean parasite density was 20,023 parasites/μL, with a trend towards higher loads in males (24,457 parasites/μL) compared to females (16,098 parasites/μL).

**Table 1:** Baseline characteristics of the study population.

Parameters	Overall N (%)	Gender	
		Male	Female
*Age category*			
N	207	108	99
<2 years	53 (25.60)	27 (25.00)	26 (26.26)
≥2 years	154 (74.40)	81 (75.00)	73 (73.74)
*Temperature (°C)*			
n	206	107	99
<37,5 °C	38 (18.45)	19 (17.76)	19 (19.19)
≥37.5°C	168 (81.55)	88 (82.24)	80 (80.81)
*Haemoglobin level (g/dL)*			
n	133	64	69
<11	108 (81.20)	52 (81.25)	56(81.16)
>11	25 (18.80)	12 (18.75)	13 (18.84)
*Parasite density*			
n	207	108	99
Geometric mean	20023	24457	16098
(95%CI)	(14997 - 26769)	(16478 - 36299)	(10463 - 24767)

### Prevalence of malaria parasite species

*P. falciparum* was the predominant species in the two detection methods used in this study. The non-*falciparum* species were detected by microscopy in 1.93%, including 1.44% (03/207) of *P. malariae* and 0.48% (01/207) of *P. ovale*. In addition to these patent cases, sub-patent infection with *P. ovale* and *P. malaria* were detected in 2.90% (06/207) and of cases 2.73 % (5/207) respectively (Table 2).

**Table 2:** Prevalence of plasmodial species by microscopy and nested PCR.

Malaria species	Diagnostic method	
	Microscopy n (%)	Nested PCR n (%)
*P. falciparum*	203 (98.07)	196 (94.69)
*P. malariae*	1 (0.48)	1 (0.48)
*P. ovale*	0(0)	1 (0.48)
*Pf+ Pm*	2 (0.97)	4(1.93)
*Pf+Po*	1 (0.48)	5 (2.42)

### Relationship between parasite density and type of infection (mono-infection or mixed infection)

There was a slightly non-significant (p=0.0583) relationship between parasite density and type of infection. Therefore, children with a *P. falciparum* mono-infection had a significantly higher geometric parasite density (21,300 parasites/μL, 95%CI: 15,800 – 28,716), than those with a mixed infection with *P. malariae* or *P. ovale* with lower densities, (6654 parasites/μL, 95%CI: 1935 – 22874) also with significant variability.

### The Effect of age, and sex on the mixed infections positivity rate

**Figure 1:**
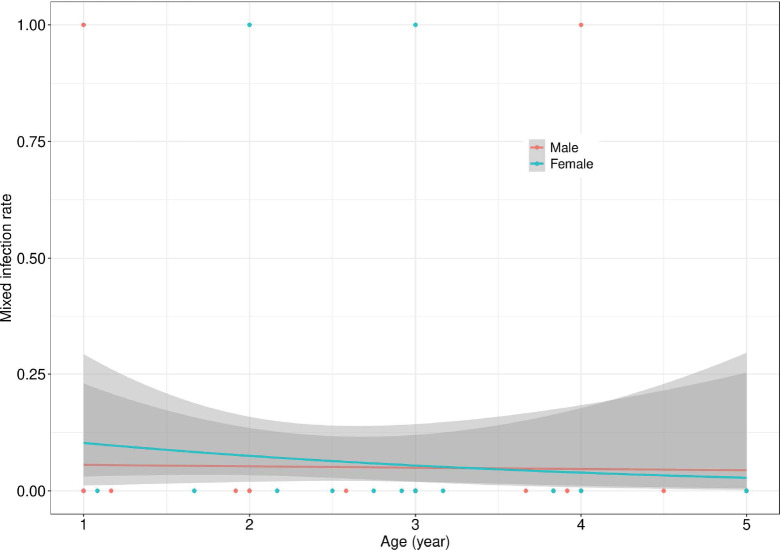
Effect of age and sex on the mixed infections positivity rate. This figure shows the positivity rate for mixed infections with a 95% confidence interval as a function of the participant's age and sex. Solid lines represent the estimated positivity rates while the shaded areas indicate the 95% confidence interval for males (dark gray) and females (light gray).

The rates of mixed infections were very low in this population, regardless of age or gender. There was no effect of gender and age on the mixed infection positivity rate (X^2^=0.16,p=0.6830, Figure 1). However, a slight decrease in the mixed infection rate was observed up to the age of 3 yrs, followed by a moderate increase up to 5 yrs.

## Discussion

This study reports the presence of *P. malariae* and *P. ovale* among children with positive diagnosis of uncomplicated malaria based on HRP2-based RDTs in the Nanoro health district of Burkina Faso. Although the prevalence of *P. malariae*, *P. ovale*, and mixed infections was low, their detection and characterisation by PCR highlights the limitations of conventional diagnostic tools, particularly rapid diagnostic tests that specifically target *P. falciparum*. The observation that PCR has the highest level of non-*falciparum* detection in this study is similar to the reports of studies conducted in high as well as low malaria settings across Africa such as Nigeria [25], Ghana [26], Ivory Coast [27], Democratic Republic of Congo [28], and Tanzania [29].

However, the prevalence of non-*falciparum* infections found in this study remains lower than that reported in other African countries for these two species (*P. malariae* and *P. ovale*) [30,[31]. These differences could be due to geographic and temporal variations as well as difference in the molecular techniques and sampling strategies.

Furthermore, the majority of infections with *P. malariae* and *P. ovale* occurred in mixed infections with *P. falciparum* as previously reported from elsewhere in sub-Saharan Africa [32,[33].

Interestingly, some cases of mono-infections with *P. malariae* or *P. ovale* were detected by PCR while HRP2-RDT results were positive. It is likely that these infections were preceded by a recently cleared *P. falciparum* infection, with residual circulating HRP2 antigen. This hypothesis is supported by previous studies showing that HRP2 antigen can persist in the blood for up to 28 days after parasite clearance [7,[18].

In this study, mixed infections exhibited lower parasite densities than mono infections with *P. falciparum*. These findings are consistent with previous studies indicating that mixed infections involving *P. falciparum* frequently present with low parasitaemia, which makes their identification more challenging [34]. This low parasitaemia may result, on the one hand, from the suppressive effect exerted by *P. falciparum* on minor species, and on the other hand, from the potentially more chronic nature of mixed infections compared to acute *P. falciparum* mono-infections. Previous studies have suggested that the presence of multiple *Plasmodium* species or genotypes in the same host may be affected by immunological and parasitic factors. For example, the host's immune response tends to initially target the dominant species or genotypes with the highest density. This favours the persistence of other species at submicroscopic levels [35,[36]. According to the density-dependent regulation model, parasitaemia of the dominant species is suppressed during co-infection, thereby maintaining a balance amongst species. However, when the density of the dominant species reaches a critical threshold, a specific immune response is triggered to control the infection. Another potential explanation of the lower parasite densities in mixed infections is the existence of competition among these species within the host [37,[38]. This would be in favour of the most competitive species while the growth of the less competitive species is reduced. However, this is influenced by species preferences. Whereas *P. malariae* and *P. ovale* infect only mature and young (reticulocytes) RBCs respectively, *P. falciparum* prefers young RBCs but is capable of invading all RBC age classes [39]. Hence, high *P. falciparum* parasite density may reduce the availability of RBCs for invasion by other species [38].

Though no significant effect of gender and age on the prevalence of mixed infections was observed, a trend toward an increase of this prevalence with age was slightly present. This observation could be explained by cumulative exposure to different *Plasmodium* species over time or by variations in immune response related to age. A study in Zambia found that children aged 2-5 yrs were at significantly higher risk of mixed malaria infections than children <2 yrs [15]. These findings suggest that age is an important factor in the dynamics of mixed infections. This is likely linked to repeated parasite exposure or the gradual development of partial immunity [40].

A limitation of this study is the cross-sectional design which does not capture temporal and seasonal variations in *Plasmodium* species distribution. Another limitation is the incapability to distinguish the specific contribution (in terms of parasite load) of each species in mixed infections. Therefore, future studies incorporating longitudinal approaches combined with quantitative PCR (qPCR) techniques would be necessary to better assess parasite load, analyse infection dynamics over time and differentiate active cases from chronic low-density carriage.

## Conclusions

The widespread use of histidine-rich protein 2 (HPR2)-based rapid diagnostic tests underestimates the malaria burden caused by non-*falciparum* species. In the context of eliminating malaria, new diagnostic tools allowing the detection of *Plasmodium* species other than *P. falciparum* must necessarily be deployed.
